# Longitudinal assessment of PCBs and chlorinated pesticides in pregnant women from Western Canada

**DOI:** 10.1186/1476-069X-4-10

**Published:** 2005-06-01

**Authors:** John Jarrell, Siu Chan, Russ Hauser, Howard Hu

**Affiliations:** 1 Department of Obstetrics and Gynecology, University of Calgary, 1430 29th ST NW, Calgary, AB T2N 2T9, Canada; 2 Department of Environmental Health, Harvard School of Public Health, Landmark Center, 3rd Floor East, 401 Park Drive, Boston, MA, 02215, USA

## Abstract

**Background:**

Maternal exposures to organochlorines prior to pregnancy are considered a risk to neonatal welfare, specifically in relation to neurocognitive functions. There is growing interest in the evaluation of maternal blood testing as a marker for fetal exposure as well as the variable geographic distribution of these priority chemicals.

**Methods:**

Three hundred and twenty-three women in the second trimester of pregnancy entered the study at a prenatal clinic providing genetic counselling information. Subjects who had an indication for genetic amniocentesis based on late maternal age were eligible to participate. Two hundred and thirty-eight completed an environmental questionnaire. A sample of amniotic fluid was taken for karyotype analysis in 323 women and blood samples during pregnancy (209), at birth (105) and from the umbilical cord (97) and breast milk (47) were also collected. These samples were tested for 29 PCB congeners and organochlorine pesticides.

**Results:**

The concentrations of PCB 153 in these media were relatively low in relation to other studies. Σ PCBs measurements in samples taken during the second trimester of pregnancy, at birth and in the umbilical cord were strongly correlated. Specific measurements of PCB 153 and PCB 180 among those subjects with completed sampling of blood samples from mothers and cord samples were significantly correlated. The concentrations of PCBs and pesticides did not differ in relation to prior spontaneous abortion history. There were no organochlorines present in the amniotic fluid at the current level of quantification.

**Conclusion:**

Pregnant women from the Western Canada region of Calgary, Alberta are exposed to relatively low concentrations of organochlorines. Measurement of maternal blood during the second trimester of pregnancy can reliably estimate the fetal exposure to PCBs. This estimate is reliable for Group 2 and 3 PCBs as well as PCB 153 and PCB 180. The amniotic fluid does not contain measurable concentrations of pesticides and PCBs under the conditions of the levels of quantification.

## Background

Polychlorinated biphenyls (PCBs) are ubiquitously present in the ecosystem and have been reported in the tissues of many animals and human population groups [1]. They are persistent and biomagnify in biota such that humans and predators at the top of the food chain experience the highest concentrations. Banned production of organochlorines in Canada and the United States in the 1970's has had an important effect in reducing exposure to these chemicals but because of the large residual quantities, release from storage and the limited rates of environmental degradation, they are still considered present in the environment. There is increasing interest in the geographic distributions of these chemicals and their potential impact on human health[2,3]

Maternal exposure to PCBs and organochlorine pesticides is an area of intense research. This is due to the potential long term effects of organochlorine exposure in the fetus and newborn[4]. Potentially severe adverse health effects during pregnancy have included preterm labor and intrauterine growth restriction in association with DDE, but the effects occurred at levels identified during the period 1959–1966[5]. These concentrations are substantially above exposure levels generally observed among current populations [6].

Postnatal adverse events have focused on the known neurotoxic effects of polychlorinated biphenyls [7]. Several studies have implicated exposure to these chemicals with impaired intellectual function in children [8-10]. Importantly the findings have demonstrated modest but significant effect sizes [10]. The potential mechanisms underlying these findings is unknown but recently dose-response relationships have been found between the concentrations of PCBs among members of the Oswego study and increases in response inhibition errors as well as reduced size of the splenium of the corpus callosum [11]. Another proposed mechanism of toxicity in the developing brain is the potential for altered thyroid function, a known contributor to impaired intelligence among newborns [12-14]. There is now evidence the actions of PCBs may reflect a direct effect of the chemical on the action of thyroid hormone in the absence of altered hormone concentrations [15]

The severity of the potential adverse central nervous system effects in children and the possibility that early intrauterine exposure to organochlorines is a determinant of impaired intelligence indicates a need for further exposure information. There is little information related to the longitudinal patterns of PCB levels in women during the pregnancy. In some cases there was no correlation between maternal and cord samples [16], although others have reported that there is a strong correlation [17]. The measurement of serum concentrations over the course of the pregnancy and delivery appears not to have been explored in detail previously although the ability to evaluate fetal exposure during pregnancy is an important objective, particularly because of the susceptibility of the developing nervous system.

This study was undertaken to define the concentrations of PCBs and organochlorine pesticides in amniotic fluid, maternal blood collected during the second trimester, and at the time of birth, cord blood and breast milk among a cohort of pregnant women from Calgary, Alberta, Canada. The inter-relationships of the concentrations in these compartments were of interest to determine if maternal blood levels measured during pregnancy could serve as a proxy for fetal exposure. Further, we were particularly interested in the concentrations of PCBs in the Calgary region compared to other studies that had evaluated neurocognitive development in local children. Finally, there was an interest in evaluating the concentrations of PCB 153 in light of its proposed use for comparing across environmental epidemiologic studies of PCB toxicity [18].

## Methods

Pregnant women attending a prenatal counselling session at Foothills Hospital in the Calgary Health Region, Calgary, Alberta were approached to participate in this project. Approval of the project was obtained from the University of Calgary Ethics Committee. Three hundred and twenty-three subjects were enrolled after their eligibility to participate was determined. They were required to be seeking genetic counselling for the purpose of age-related indications and not to have another reason for such counselling. Subjects were also required to be 35 years of age at the time of entry

Of those agreeing to participate, two hundred and thirty-eight completed an environmental questionnaire. A sample of amniotic fluid was available from three hundred and twenty-three women. A blood sample at the same time as the amniocentesis during the second trimester of pregnancy was available from 209 women. A blood sample at the time of delivery was made available in 105 women. Cord blood samples were collected from 97 women and a sample of breast milk was collected from 47 women after birth and during the puerperium from home. Clinical information related to the pregnancy and delivery was collected at the three Calgary hospitals and three hospitals outside the Calgary health region. All subjects were given the special tubes and phlebotomy supplies to submit various blood and breast milk samples and contacted on a regular basis for the completion of the sampling. The nursing staff on each delivery suite was oriented to the project to gain maximal compliance with the study.

The blood samples were collected by the regional laboratory in specially prepared glass tubes, centrifuged and the serum transferred to the Centre for Toxicology, University of Calgary for analysis. A similar approach was used for amniotic fluid. It should be noted that subjects were not required to fast for the blood samples, either during pregnancy or at birth.

The samples were assayed at the Centre for Toxicology, University of Calgary. They underwent sample clean-up and extraction by solid phase extraction techniques. Details of the methods have been previously reported [19,20]The PCBs and most pesticides were measured with GC/negative chemical ionization spectrometry (GC/NCIMS). Some pesticides were measured with GC/electron ionization mass spectrometry (GC/EIMS). The Centre participated in the Intercomparison Program for Toxicological Analysis in Biological Materials, University of Erlangen-Nuremberg, and the performance had been good

In amniotic fluid the PCBs measured were as follows: PCB- 70, 74, 77, 87, 99, 101, 105, 118, 128, 138, 151, 153, 156, 169, 170, 180, 183, 187, 191, 194, 205, 206, 208, 209. The pesticides measured were: Aldrin, p, p'-DDE, p, p'-DDT, dieldrin, endosulfan I and II, endrin, heptachlor, hexachlorobenzene, hexachlorocyclohexanes, α-,β-,γ-, hexachloroethane, methoxychlor, mirex, pentachlorobenzene, 1,2,3-trichlorobenzene, 1,2,4-trichlorobenzene, 1,2,3,4-tetrahlorobenzene. These analytes were also measured in serum and breast milk with some minor changes

The limit of quantification (LOQ) varied among analytes as well as matrices. For example, the LOQ for most PCBs was 0.01 ng/ml in all matrices. The LOQ for most other industrial chemicals was 0.05 ng/ml, however there were exceptional situations. For example, the LOQ for p, p'-DDT was 0.5 ng/ml. In order to evaluate the measurements of Σ PCBs and Group 2 and 3 PCBs, 1/2 of the level of detection was used in analysis. This may result is some bias in the analysis as only PCB 153, 138, 180 and 170 had detection rates >90% in maternal blood during pregnancy. There were similar patterns of detection rates for the PCBs 153, 138 and 103 in maternal blood at birth and PCBs 153 in cord blood and PCB 118, 153, 138, 187, 183, 156, 180, 170 and 194 in breast milk. The presence of bias in the use of this technique is appreciated. Other approaches such as the use of designation of non-detected samples as zero values was not used because the values are called non-ignorable data that may also result bias if the samples are simply excluded. Alternative imputation analysis approaches may provide more accurate methods for pregnancy related PCBs but will need to be confirmed in comparison to other studies [21].

The lipid content of serum was calculated by the sum of cholesterol, triglycerides and phospholipids. These analytes were measured by enzymatic methods using a chemistry analyzer. The lipid content of milk was measured by a gravimetric method.

### Statistical analysis

The data from the clinical charts, analysis of chemical results and the environmental questionnaires were assembled in SPSS. Only normally distributed data were evaluated using parametric analysis (paired t-test and Pearson Correlation). Parametric analysis was undertaken wherever possible. The individual analytes were not normally distributed and were normalized by logarithmic function and adjusted to account for non-detection of samples. All PCB concentrations were highly skewed. Samples were corrected for non-detection (0.5*level of quantification), lipid adjusted and log transformed for a normal distribution. Because the relationships of maternal blood to cord blood are key in this study and the fetal blood is lower in lipid content, PCBs levels were evaluated as ng/ml and as ng/g lipid. Specific analysis was done on PCB 153 because of the relatively high concentrations observed. Samples also were aggregated by measuring Σ PCBs as well as Σ PCBs in Group 2 and Group 3 of those proposed by Wolff et al. [22]. For this study, Group 2 included PCBs 74, 118, 156, 138 and 170. Group 3 included PCBs 99, 153, 180 and 183. Data concerning the concentrations of HCB and DDE were treated in a similar fashion for non-detection and normalization.

To explore changes in the concentrations in pregnancy, paired t-tests were undertaken between all possible groupings of samples on the normalized data. Although there were sufficient pairings to evaluate differences in the pair groups, it is important to note these comparisons do not exactly reflect the same cohort over the four periods of study because of missing data. In order to evaluate the correlation of the results, and bearing in mind the desire to study the maternal blood during pregnancy and at birth as a potential marker of fetal exposure, the non-lipid adjusted samples were normalized for comparison purposes. The non-detection samples were adjusted to reflect 0.5 the level of quantification.

To evaluate the potential association of exposures to spontaneous abortion, the concentrations were assessed in relation to clinical history by one way analysis of variance.

## Results

### Demographics

The subjects were all from the Calgary Health Region, located in southern Alberta, Canada during the period from an entry in 2001 to 2003 when the last delivery occurred. A total of 323 subjects were approached and 315 subjects completed the consent form and formally entered into the study. From this group, 308 provided samples of amniotic fluid for karyotype studies as well as the measurement of the organochlorines. The balance of subjects decided not to have the amniocentesis undertaken based upon information from the Genetics Counselling Clinic. There were 209 blood samples collected during the second trimester around the time of the amniocentesis. There were 105 blood samples analyzed from the mothers at the time of giving birth. There were 97 samples of cord blood and 47 samples of breast milk collected. The breast milk samples were collected at varying times in the postpartum period.

There were 203 pairs of amniotic fluid and maternal blood during the time of amniocentesis and 85 pairs of samples that were obtained from maternal blood at birth and cord samples. There were 23 subjects in which all samples were collected in all tissues compartments.

The demographic and reproductive histories of the subjects are presented in Table 1. Consistent with the inclusion criteria, the age of the subjects was 39.0+/- 0.1 (Mean+/- SEM). During the previous year, 15.1% of the subjects identified themselves as smokers and 8% continued to smoke during the pregnancy. Most of these smokers reported smoking less than 20 cigarettes per day (see Additional file: 1).

A specific diet was reported by 66 subjects including weight loss (13.4%), sodium free (3.4%), fat free (8.4%), diabetic (0.4%), weight gain (0.4%) and vegetarian (1.7%). Eighty five individuals reported a significant weight loss on the preceding year and the average amount was reported to be 7.3 pounds.

Prior medical history was significant for the regular use of prescription medication (27%), Anesthetic exposure (18%), and infertility (12%). There was an infrequent rate of prior cancer (2.1%), skin disease (2.9 %) and liver disease (1.7%).

The most common chemical exposure in the previous year was to paint (50.5%) and solvents (32.8%). There was a reported exposure of this cohort to pesticides in 22%, herbicides 22%, fungicides 5.9% and dry cleaning chemicals 6.5%.

Of the cohort, there were only 36 primigravidas. Sixteen percent reported one induced abortion and 6% had had 2 or more. There were 21% of the subjects with one, 6.3% with two and 6% with three or more spontaneous abortions. Two percent reported an ectopic pregnancy.

Of the pregnancies under review, there were 291 births, of which there was a complication rate of pregnancy of pregnancy induced hypertension in 8.2%, premature labor in 3.4%, gestational diabetes in 5.2% a birth weight less than 2500 g in 4.8% and a Caesarean section rate of 27%.

The sex ratio of the cohort of infants was 1.13 (male/female). Karyotype indicated two cases of Klinefelter syndrome and 2 cases of trisomy that proceeded to delivery. Other anomalies resulted in pregnancy termination.

#### Concentrations of the Organochlorine Chemicals

##### Amniotic Fluid

None of the PCBs or pesticides was measurable in the amniotic fluid. It should be noted the amniotic fluid measurement was done after centrifugation and the amniocytes removed for karyotyping.

##### Total PCBs and Pesticides

The concentrations of all available samples of PCB 153, Σ PCBs, Group 2 and Group 3 PCBs and the pesticides DDE and HCB (lipid adjusted) are presented in Additional file: 2. Statistical analysis was performed for Σ PCB samples using a paired t-test of normalized data. In relation to ΣPCBs, samples collected from women during pregnancy did not differ from those collected at birth (p > 0.05) but the levels in maternal blood were higher than samples from the cord and breast milk (p < 0.001). Breast milk was found to be higher than cord blood ΣPCBs (p = 0.008) all tests after lipid-adjustment.

A similar pattern was identified for Group 2 and Group 3 PCBs. There were no differences between blood collected during pregnancy and the samples taken at birth (n.s.). There were higher concentrations in blood taken during pregnancy and at birth over cord blood (p < 0.01) and breast milk (p < 0.01). Breast milk was higher than cord blood for Group 2 (p < 0.05) but not Group 3 PCBs.

PCB 153 concentrations were not different between maternal blood during pregnancy and at birth although they were all significantly higher than breast milk.

This increase in the concentrations of PCBs in the cord blood samples was potentially a consequence of the relatively low concentrations of lipid in the fetal circulation. The levels of non-lipid adjusted levels of PCBs are shown in Additional file: 2. The lipid concentrations of the tissue compartments were as follows (mean +/- S dev.): maternal blood during pregnancy – 0.71+/-0.13, at birth – 0.74+/-0.16, cord blood- 0.21+/-0.06 and breast milk – 2.83+/-1.33 g/100 ml plasma.

The pattern of PCBs among non-lipid adjusted groups is shown in Additional file: 2. There was no difference in the maternal samples during pregnancy compared to the cord blood samples. There were significantly higher levels of both Groups in maternal blood during pregnancy and at birth compared to cord blood, consistent with the Σ PCB level pattern (p < 0.001)

The concentrations of pesticides are presented in Additional file: 2. There were no differences in lipid adjusted HCB concentrations of maternal blood during pregnancy and at birth when compared to cord blood (n.s.) There were significantly lower concentrations of lipid adjusted HCB in breast milk when compared to all other groups (p < 0.001). The same pattern of result was observed for lipid adjusted DDE samples.

Without lipid adjustment there was a significant difference in HCB in maternal blood during pregnancy and at birth compared to cord blood (p < 0.001) (see Additional file: 2). HCB during pregnancy and at birth were significantly less than breast milk samples without lipid adjustment (p < 0.001). The same pattern was observed for non-lipid adjusted DDE samples.

##### PCB Correlations

The relationships of the Σ PCBs are closely correlated in all of the tissues as indicated in Table 3. There are several notable relationships with very high correlation coefficients. Non-lipid adjusted, normalized Σ PCBs during pregnancy were found to highly correlate with levels at birth (r = 0.770, p < 0.01) and in cord blood (r = 0.498, p < 0.01) but not with breast milk (n.s.). Σ PCBs taken at birth also correlated well with cord blood (p < 0.001) but not with breast milk PCBs.

**Table 3:** Correlation of ΣPCBs In Human Tissues

		**ΣPCB DP**	**ΣPCB AB**	**ΣPCB CB**	**ΣPCB BM**
**ΣPCB DP**	**r**	1.000	0.759	0.357	0.504
	**p**		0.000	0.001	0.001
	**n**		97.000	91.000	40.000

**ΣPCB AB**	**r**		1.000	0.600	0.325
	**p**			0.000	0.075
	**n**			87.000	31.000

**ΣPCB CB**	**r**			1.000	-0.012
	**p**				0.954
	**n**				27.000

There were significant correlations between Group 2 and 3 PCBs (Additional file: 3). The most significant correlations were between groups but within tissue compartments. Group 2 PCBs of maternal blood during pregnancy correlated most strongly with Group 3 PCBs of maternal blood during pregnancy (r = 0.755, p < 0.01) but also strongly correlated with Group 2 PCBs at birth (r = 0.628, p < 0.01) and Group 3 PCBs at birth (r = 0.717, p < 0.001); Group 3 PCBs of maternal blood during pregnancy also correlated with Group 2 and 3 at birth and Group 2 and 3 cord blood (all p < 0.001). The Group 2 PCBs at birth correlated well with Group 2 and 3 cord blood cord (r = 0.603, p < 0.001 and r = 0.783, p < 0.001 respectively); Group 3 PCBs at birth also correlated with Group 2 and 3 cord blood (all p < 0.001). There was no correlation between breast milk and the other values.

To explore more rigorously the relationships of PCBs between compartments PCB 153 and PCB 180 were evaluated among all subjects with completed collection of maternal and fetal blood samples. These PCBs were selected because of the relatively high rate of detection of analytes. Cord blood PCB 153 was significantly correlated with maternal blood during pregnancy (r = 0.691, p < 0.001) and maternal blood at birth (r = 0.905, p < 0.001). Cord blood PCB 180 was also significantly correlated with maternal blood during pregnancy (r = 0.511, p < 0.001) and maternal blood at birth (r = 0.830, p < 0.001).

The correlations between the tissue compartments and HCB and DDE are presented in Additional file: 4. Specifically, there were significant correlations for both HCB and DDE between both the samples during pregnancy and at birth when compared to cord blood (all p < 0.001).

##### Clinical Variables and Concentrations of PCBs and Pesticides

There were no differences in the concentrations of PCBs or pesticides among women reporting one or two prior spontaneous abortions compared to those individuals who had had no spontaneous abortion. It should be noted this was not the primary objective of the study and the clinical outcomes are part of secondary analysis. There were no significant differences in concentrations of PCBs and pesticides among the subjects reporting medical conditions with the exception that those individuals reporting infertility had significantly higher concentrations of HCB. There were no differences in these concentrations among women reporting exposure to herbicides, pesticides, fungicides or insecticides compared to those reporting no such exposure in the previous year. Similarly, there was no significant association of a particular diet (weight loss, diabetic, salt free, low fat) with the measurement of PCB and pesticides in this cohort of subjects. Although there was a dose related increase in the mean concentrations of Σ PCBs in maternal blood during pregnancy in relation to fish consumption, the relationship was not significant.

## Discussion

The results of this study are reassuring and support the observations that indicate levels of PCBs are relatively low in western Canada compared to other studies in previous years among pregnant women in different countries or regions of Canada. Specifically, using PCB 153 as a marker as recommended [23], it would appear the women in Calgary are at the lower range of values among studies that evaluated neurocognitive outcomes of children. The meta analysis of ten studies indicated a range of the 25^th ^to the 75^th ^percentiles of PCB 153 to range from approximately 20 to 800 ng/g lipid [18]. In this study, the arithmetic mean concentrations of PCB 153 were 21.16, 25.71 and 18.22 ng/g lipid in the maternal blood during pregnancy, at birth and cord blood respectively, indicating a relatively low exposure level in relation to the previous reports.

There is evidence that the maternal serum during the second trimester of pregnancy can be considered a biomarker for the exposure of the fetus *in utero *as determined by cord blood levels and the significant correlations among these tissue compartments. These observations have been made previously at higher PCB concentrations [3]

The concentrations of this study are consistent with other reports of the concentrations of PCB 153, HCB and DDE in the cord samples. Specifically, there is close agreement with the findings in southern Quebec, Canada and New Bedford, USA for all three chemicals measured on a wet weight basis [24]. Additionally the combination of PCB 138, 153 and 180 in the New Bedford study was approximately twice the concentrations of the same PCBs in the current study (mean, median and standard deviation: 0.84, 0.6, 1.14 ng/ml respectively), and agreed more closely with the Σ PCBs which contained PCBs 74, 118, 138, 153, 187, 183, 156, 180, 170. These differences may reflect a greater exposure in New Bedford than in southern Alberta. The differences in cord concentrations from other reports of higher levels may be a reflection of the declining concentrations in recent years since the chemicals were banned [25-28]. Decreases in tissue concentrations have been reported in Northern Canada between 1994 and 2001 [29]. The lower concentrations may also reflect the findings of a population study of healthy women attending a genetics counselling service that was unselected for dietary intake of foods containing PCBs [30].

Absence of these chemicals in the amniotic fluid at the level of quantification was observed but not unanticipated because of the low lipid concentration, an assessment of the amniocytes may have isolated analytes [31, 32]. In this study the cells were not available owing the need for karyotyping.

There were a number of important findings from this cohort that again indicate the relatively low concentrations did not exercise a measurable biological effect of the prior health of the women. For example, there were no differences in the concentrations of PCBs or pesticides among women reporting prior spontaneous abortion. The sample size may be too small to detect a significant increase. The higher concentrations of HCB during pregnancy among those reporting infertility may simply reflect the lack of prior pregnancy-related loss of body burden and is not interpreted as a causal function of infertility. There were no significant differences in the concentrations of PCBs and pesticides among the subjects reporting current or previous serious medical conditions or exposure to pesticides, fungicides, herbicides or other common chemicals such as dry cleaning fluid in the previous year. Similarly, there was no significant association of diet with the measurement of Σ PCB and pesticides in this cohort of subjects. Although there was an increase in the mean concentrations of Σ PCBs in relation to daily fish consumption, the relationship of diet was not significant.

## Conclusion

In summary, it would appear that pregnant subjects from Calgary Alberta are exposed to PCBs and pesticides as are most individuals but the extent of exposure appears to be low in relation to other published studies from other areas of Canada or other countries. There is no evidence of exposure to pesticides or PCBs in the amniotic fluid under the current measurement limitations of quantification. Measurement of maternal blood during the second trimester of pregnancy reliably estimates the maternal blood levels at birth as well as fetal exposure to PCBs and organochlorines as measured by cord blood at these relatively low levels. The evaluation of subjects who had complete blood sampling indicated specifically that the cord blood PCB 153 and PCB 180 are reliably predicted by a measure of maternal blood during the second trimester of pregnancy.

## List of Abbreviations

DP: maternal blood taken during the first trimester of pregnancy

AB: Maternal blood taken at the time of birth

C Blood taken from the cord at birth

BM: Breast milk

DDE: Dichlorodiphenyldichloroethylene

HCB: Hexachlorobenzene

DDT: Dichlorodiphenyltrichloroethane

PCBs: Polychlorinated biphenyls

PCB 70 2,3',4',5-Tetrachlorobiphenyl

PCB 74 2,4,4',5-Tetrachlorobiphenyl

PCB 77 3,3',4,4'-Tetrachlorobiphenyl

PCB 87 2,2'3,4,5'-Pentachlorobiphenyl

PCB 99 2,2',4,4'5-Pentachlorobiphenyl

PCB 101 2,2'4,5,5'-Pentachlorobiphenyl

PCB 105 2,3,3',4,4'-Pentachlorobiphenyl

PCB 118 2,3',4,4',5-Pentachlorobiphenyl

PCB 128 2,2',3,3',4,4'-Hexachlorobiphenyl

PCB 138 2,2',3,4,4',5'-Hexachlorobiphenyl

PCB 151 2,2',3,5,5'6-Hexachlorobiphenyl

PCB 153 2,2',4,4',5,5'-Hexachlorobiphenyl

PCB 156 2,3,3'4,4',5-Hexachlorobiphenyl

PCB 169 3,3'4,4',5,5'-Hexachlorobiphenyl

PCB 170 2,2',3,3',4,4',5-Heptachlorobiphenyl

PCB 180 2,2',3,4,4',5,5'-Heptachlorbiphenyl

PCB 183 2,2'3,4,4'5',6-Heptachlorbiphenyl

PCB 187 2,2',3,4',5,5',6-Heptachlorbiphenyl

PCB 191 2,3,3',4,4',5',6-Heptachlorbiphenyl

PCB 194 2,2',3,3',4,4'5,5'-Octachlorbiphenyl

PCB 205 2,3,3',4,4',5,5',6-Octachlorobiphenyl

PCB 206 2,2',3,3',4,4',5,5',6-Nonachlorbiphenyl

PCB 208 2,2',3,3',4,5,5',6,6'-Nonachlorobiphenyl

PCB 209 Decachlorobiphenyl

## Competing interests

The author(s) declare that they have no competing interests.

## Authors' Contributions

JJ was original co-investigator and undertook patient recruitment, clinical data acquisition and data analysis in relation to the analysis of human tissue samples, and drafted the article. SC was principal investigator of the original proposal, undertook sample measurement and analysis and participated in revising the draft for consideration; RH participated in data analysis and revisions of the article; HH participated in data analysis and revisions of the article. All authors read and approved the final manuscript.

## Supplementary Material

Additional file 1 A “.doc file” describing the characteristics of the women enrolled in the study. Click here for file

Additional file 2 A “.doc file” that describes concentrations of the organochlorines isolated from the women during the study period. Click here for file

Additional file 3 A “.doc file”  describes the strength of the comparisons of the levels of certain groups of PCBs  in the women’s tissues. Click here for file

Additional file 4 A “.doc file that describes the strength of the comparisons of the levels of certain pesticides in women’s tissues. Click here for file
