# Supplementary material for: Longitudinal assessment of PCBs and chlorinated pesticides in pregnant women from Western Canada
Source: Environ Health. 2005 Jun 1;4:10. doi: 10.1186/1476-069X-4-10 (PMC1190201; doi:10.1186/1476-069X-4-10)
Supplement: Additional file 1 — A “.doc file” describing the characteristics of the women enrolled in the study. [file 1476-069X-4-10-S1.doc]

| **Additional File 1. Demographics and Reproductive History of Study Population** | | | | |
| --- | --- | --- | --- | --- |
| **Demographics** | | | | |
|  | **N** | **Minimum** | **Maximum** | **Mean** |
| **Approached** | 323 |  |  |  |
| **Entered** | 315 |  |  |  |
| **Age** |  | 35 | 45 | 29 |
|  |  | |  | |
| **Gravida** | **N** | | **%** | |
| 1 | 69 | | 21.6 | |
| 2 | 92 | | 28.8 | |
| 3 | 71 | | 22.2 | |
| >3 | 87 | | 27.4 | |
| **Para** | **N** | | **%** | |
| 0 | 113 | | 35.6 | |
| 1 | 135 | | 42.5 | |
| 2 | 48 | | 15.1 | |
| >2 | 21 | | 6.5 | |
| **Reproductive History** | | | | |
|  | **Frequency** | | **%** | |
| **Preterm Birth** | | | | |
| **Yes** | 14 | | 4.4 | |
| **No** | 302 | | 95.6 | |
| **Stillbirth** | | | | |
| **Yes** | 1 | | 0.3 | |
| **No** | 317 | | 99.6 | |
| **Therapeutic Abortion** | | | | |
| **0** | 242 | | 76.8 | |
| **1** | 57 | | 18.0 | |
| **>1** | 16 | | 3.5 | |
| **Spontaneous Abortion** | | | | |
| **0** | 205 | | 64.8 | |
| **1** | 69 | | 21.8 | |
| **2** | 30 | | 9.4 | |
| **>2** | 12 | | 3.7 | |
| **Living Children** | | | | |
| **0** | 114 | | 36.1 | |
| **1** | 133 | | 42.2 | |
| **2** | 47 | | 14.9 | |
| **>2** | 21 | | 6.5 | |
| **Smoker last year** | | | | |
| **Yes** | 36 | | 15.2 | |
| **No** | 202 | | 84.8 | |
| **Current Smoker** | | | | |
| **Yes** | 19 | | 7.9 | |
| **No** | 219 | | 92.1 | |
| **Cigarettes per Day** | | | | |
| **0** | 218 | | 91.5 | |
| **>1** | 20 | | 8.5 | |
| **1-10** | 14 | | 5.8 | |
| **>10** | 6 | | 2.5 | |
| **Sex of Infant** | | | | |
| **Male** | 167 | | 52.1 | |
| **Female** | 148 | | 47.9 | |
