# Supplementary material for: Longitudinal assessment of PCBs and chlorinated pesticides in pregnant women from Western Canada
Source: Environ Health. 2005 Jun 1;4:10. doi: 10.1186/1476-069X-4-10 (PMC1190201; doi:10.1186/1476-069X-4-10)
Supplement: Additional file 2 — A “.doc file” that describes concentrations of the organochlorines isolated from the women during the study period. [file 1476-069X-4-10-S2.doc]

**Additional File 2. Concentrations of PCBs and Pesticides During Pregnancy, At Birth, in Cord Blood and Breast Milk**

| **A. Lipid Adjusted (ng/g lipid)** | | | | | | **B. Wet Weight (ng/ml)** | | | |
| --- | --- | --- | --- | --- | --- | --- | --- | --- | --- |
| **Chemical** | **N** | **Mean** | **Min** | **Max** | **Geometric mean** | **Mean** | **Min** | **Max** | **Geometric mean** |
| **ΣPCBs** |  |  |  |  |  |  |  |  |  |
| **During Pregnancy** | 209 | 75.28 | 13.75 | 432.43 | 61.68 | 0.78 | 0.43 | 3.34 | 0.73 |
| **At Birth** | 105 | 80.99 | 11.70 | 1368.68 | 59.64 | 0.72 | 0.36 | 4.91 | 0.65 |
| **Cord Blood** | 97 | 131.85 | 55.83 | 515.31 | 122.34 | 0.25 | 0.16 | 1.88 | 0.23 |
| **Breast Milk** | 47 | 38.20 | 11.95 | 125.30 | 33.25 | 0.25 | 0.37 | 4.85 | 1.11 |
|  |  |  |  |  |  |  |  |  |  |
| **PCB 153** |  |  |  |  |  |  |  |  |  |
| **During Pregnancy** | 209 | 21.16 | 0.89 | 156.25 | 16.70 | 0.15 | 0.01 | 1.04 | 0.12 |
| **At Birth** | 105 | 25.71 | 2.60 | 522.90 | 17.60 | 0.17 | 0.01 | 1.83 | 0.13 |
| **Cord Blood** | 97 | 18.22 | 7.58 | 163.90 | 14.50 | 0.04 | 0.01 | 0.59 | 0.03 |
| **Breast Milk** | 47 | 12.19 | 2.69 | 38.24 | 10.52 | 0.32 | 0.23 | 0.99 | 0.26 |
|  |  |  |  |  |  |  |  |  |  |
| **Group 2** |  |  |  |  |  |  |  |  |  |
| **During Pregnancy** | 209 | 26.67 | 3.09 | 150.00 | 20.14 | 0.23 | 0.11 | 1.30 | 0.20 |
| **At Birth** | 105 | 29.90 | 3.25 | 484.33 | 20.92 | 0.24 | 0.10 | 1.72 | 0.21 |
| **Cord Blood** | 97 | 70.61 | 28.33 | 236.13 | 66.18 | 0.13 | 0.08 | 0.86 | 0.12 |
| **Breast Milk** | 47 | 17.30 | 4.55 | 84.55 | 14.19 | 0.62 | 0.10 | 1.98 | 0.52 |
|  |  |  |  |  |  |  |  |  |  |
| **Group 3** |  |  |  |  |  |  |  |  |  |
| **During Pregnancy** | 209 | 38.32 | 3.57 | 262.16 | 30.25 | 0.3 | 0.07 | 1.94 | 0.26 |
| **At Birth** | 105 | 44.27 | 4.55 | 804.35 | 31.48 | 0.33 | 0.08 | 2.84 | 0.27 |
| **Cord Blood** | 97 | 48.29 | 22.73 | 259.74 | 42.58 | 0.1 | 0.05 | 0.95 | 0.08 |
| **Breast Milk** | 47 | 23.67 | 8.29 | 68.53 | 20.70 | 0.62 | 0.10 | 1.98 |  |
|  |  |  |  |  |  |  |  |  |  |
| **HCB** |  |  |  |  |  |  |  |  |  |
| **During Pregnancy** | 209 | 17.23 | 2.81 | 159.04 | 14.33 | 0.12 | 0.03 | 1.32 | 0.10 |
| **At Birth** | 105 | 18.28 | 3.25 | 177.16 | 14.37 | 0.13 | 0.03 | 0.62 | 0.11 |
| **Cord Blood** | 97 | 15.60 | 7.58 | 100.00 | 13.91 | 0.03 | 0.03 | 0.13 | 0.02 |
| **Breast Milk** | 37 | 6.06 | 2.55 | 11.25 | 5.63 | 0.16 | 0.03 | 0.36 | 0.14 |
|  |  |  |  |  |  |  |  |  |  |
| **DDE** |  |  |  |  |  |  |  |  |  |
| **During Pregnancy** | 200 | 153.23 | 3.68 | 1625.00 | 109.85 | 1.06 | 0.30 | 10.40 | 0.76 |
| **At Birth** | 105 | 164.09 | 2.55 | 1392.19 | 110.93 | 1.1 | 0.30 | 8.90 | 0.76 |
| **Cord Blood** | 97 | 121.79 | 10.42 | 1073.33 | 76.59 | 0.25 | 0.03 | 2.00 | 0.16 |
| **Breast Milk** | 37 | 101.90 | 16.13 | 989.05 | 68.06 | 2.57 | 0.15 | 20.80 | 1.63 |
