# Supplementary material for: Longitudinal assessment of PCBs and chlorinated pesticides in pregnant women from Western Canada
Source: Environ Health. 2005 Jun 1;4:10. doi: 10.1186/1476-069X-4-10 (PMC1190201; doi:10.1186/1476-069X-4-10)
Supplement: Additional file 3 — A “.doc file” describes the strength of the comparisons of the levels of certain groups of PCBs in the women’s tissues. [file 1476-069X-4-10-S3.doc]

| **Additional File 4. Correlation of Group 2 and 3 PCBs in Human Tissues** | | | | | | | | | |
| --- | --- | --- | --- | --- | --- | --- | --- | --- | --- |
|  | | **Group 2** | **Group 3** | **Group 2** | **Group 3** | **Group 2** | **Group 3** | **Group 2** | **Group 3** |
|  |  | **DP** | **DP** | **AB** | **AB** | **CB** | **CB** | **BM** | **BM** |
| **Group 2** | r | 1.000 | 0.798 | 0.642 | 0.649 | 0.211 | 0.325 | 0.442 | 0.500 |
| **DP** | p | . | 0.000 | 0.000 | 0.000 | 0.045 | 0.002 | 0.004 | 0.001 |
|  | n |  | 209 | 97 | 97 | 91 | 91 | 40 | 40 |
| **Group 3** | r | 0.798 | 1.000 | 0.753 | 0.776 | 0.284 | 0.431 | 0.307 | 0.629 |
| **DP** | p | 0.000 | . | 0.000 | 0.000 | 0.006 | 0.000 | 0.054 | 0.000 |
|  | n | 209 |  | 97 | 97 | 91 | 91 | 40 | 40 |
| **Group 2** | r | 0.642 | 0.753 | 1.000 | 0.975 | 0.518 | 0.647 | 0.277 | 0.265 |
| **AB** | p | 0.000 | 0.000 | . | 0.000 | 0.000 | 0.000 | 0.132 | 0.150 |
|  | n | 97 | 97 |  | 105 | 87 | 87 | 31 | 31 |
| **Group 3** | r | 0.649 | 0.776 | 0.975 | 1.000 | 0.514 | 0.634 | 0.156 | 0.426 |
| **AB** | p | 0.000 | 0.000 | 0.000 | . | 0.000 | 0.000 | 0.403 | 0.017 |
|  | n | 97 | 97 | 105 |  | 87 | 87 | 31 | 31 |
| **Group 2** | r | 0.211 | 0.284 | 0.518 | 0.514 | 1.000 | 0.802 | 0.027 | -0.023 |
| **CB** | p | 0.045 | 0.006 | 0.000 | 0.000 | . | 0.000 | 0.894 | 0.908 |
|  | n | 91 | 91 | 87 | 87 |  | 97 | 27 | 27 |
| **Group 3** | r | 0.325 | 0.431 | 0.647 | 0.634 | 0.802 | 1.000 | -0.044 | -0.198 |
| **CB** | p | 0.002 | 0.000 | 0.000 | 0.000 | 0.000 | . | 0.826 | 0.323 |
|  | n | 91 | 91 | 87 | 87 | 97 |  | 27 | 27 |
| **bm_2** | r | 0.442 | 0.307 | 0.277 | 0.156 | 0.027 | -0.044 | 1.000 | 0.777 |
|  | p | 0.004 | 0.054 | 0.132 | 0.403 | 0.894 | 0.826 | . | 0.000 |
|  | n | 40 | 40 | 31 | 31 | 27 | 27 |  | 47 |
| **bm_3** | r | 0.500 | 0.629 | 0.265 | 0.426 | -0.023 | -0.198 | 0.777 | 1.000 |
|  | p | 0.001 | 0.000 | 0.150 | 0.017 | 0.908 | 0.323 | 0.000 | . |
|  | n | 40 | 40 | 31 | 31 | 27 | 27 | 47 |  |
