# Supplementary material for: Longitudinal assessment of PCBs and chlorinated pesticides in pregnant women from Western Canada
Source: Environ Health. 2005 Jun 1;4:10. doi: 10.1186/1476-069X-4-10 (PMC1190201; doi:10.1186/1476-069X-4-10)
Supplement: Additional file 4 — A “.doc file that describes the strength of the comparisons of the levels of certain pesticides in women’s tissues. [file 1476-069X-4-10-S4.doc]

| **Additional File 5. Correlations of Pesticides HCB and DDE** | | | | | | | | | |
| --- | --- | --- | --- | --- | --- | --- | --- | --- | --- |
|  | | **HCB** | | | | **DDE** | | | |
|  |  | **DP** | **AB** | **CB** | **BM** | **DP** | **AB** | **CB** | **BM** |
| **HCB** | **r** | 1.000 | 0.540 | 0.179 | 0.404 | 0.269 | 0.211 | -0.020 | 0.022 |
| **DP** | **p** | . | 0.000 | 0.089 | 0.020 | 0.000 | 0.038 | 0.849 | 0.903 |
|  | **n** |  | 97 | 91 | 33 | 209 | 97 | 91 | 33 |
| **HCB** | **r** |  | 1.000 | 0.269 | 0.627 | 0.187 | 0.342 | 0.062 | 0.109 |
| **AB** | **p** |  | . | 0.012 | 0.001 | 0.067 | 0.001 | 0.568 | 0.595 |
|  | **n** |  |  | 87 | 26 | 97 | 97 | 87 | 26 |
| **HCB** | **r** |  |  | 1.000 | 0.358 | -0.135 | -0.132 | 0.260 | -0.189 |
| **CB** | **p** |  |  | . | 0.102 | 0.201 | 0.237 | 0.010 | 0.400 |
|  | **n** |  |  |  | 22 | 91 | 82 | 97 | 22 |
| **HCB** | **r** |  |  |  | 1.000 | 0.195 | 0.373 | 0.134 | 0.190 |
| **BM** | **p** |  |  |  | . | 0.278 | 0.073 | 0.551 | 0.260 |
|  | **n** |  |  |  |  | 33 | 24 | 22 | 37 |
| **DDE** | **r** |  |  |  |  | 1.000 | 0.873 | 0.591 | 0.987 |
| **DP** | **p** |  |  |  |  | . | 0.000 | 0.000 | 0.000 |
|  | **n** |  |  |  |  |  | 97 | 91 | 33 |
| **DDE** | **r** |  |  |  |  |  | 1.000 | 0.544 | 0.904 |
| **AB** | **p** |  |  |  |  |  | . | 0.000 | 0.000 |
|  | **n** |  |  |  |  |  |  | 82 | 24 |
| **DDE** | **r** |  |  |  |  |  |  | 1.000 | 0.962 |
| **CB** | **p** |  |  |  |  |  |  | . | 0.000 |
|  | **n** |  |  |  |  |  |  |  | 22 |
